# False Alarms in Wearable Cardioverter Defibrillators—A Relevant Issue or an Insignificant Observation

**DOI:** 10.3390/jcm13247768

**Published:** 2024-12-19

**Authors:** Phi Long Dang, Philipp Lacour, Abdul Shokor Parwani, Felix Lucas Baehr, Uwe Primessnig, Doreen Schoeppenthau, Henryk Dreger, Nikolaos Dagres, Gerhard Hindricks, Leif-Hendrik Boldt, Florian Blaschke

**Affiliations:** 1 Department of Cardiology, Angiology and Intensive Care Medicine, Deutsches Herzzentrum der Charité (DHZC), Campus Virchow-Klinikum, Augustenburger Platz 1, 13353 Berlin, Germany; phi-long.dang@charite.de (P.L.D.); abdul.parwani@dhzc-charite.de (A.S.P.); doreen.schoeppenthau@dhzc-charite.de (D.S.); henryk.dreger@dhzc-charite.de (H.D.); leif-hendrik.boldt@charite.de (L.-H.B.); 2 Study Center Berlin, IB University of Health and Social Sciences, 12683 Berlin, Germany; 3 DZHK (German Centre for Cardiovascular Research), 10785 Berlin, Germanygerhard.hindricks@dhzc-charite.de (G.H.); 4 Department of Cardiology, Angiology and Intensive Care Medicine, Deutsches Herzzentrum der Charité (DHZC), Campus Charité Mitte, Charitéplatz 1, 10117 Berlin, Germany

**Keywords:** wearable cardioverter defibrillator, sudden cardiac death, artifact sensing, episode misclassification

## Abstract

**Background:** The wearable cardioverter defibrillator (WCD) has emerged as a valuable tool used for temporary protection from sudden cardiac death. However, since the WCD uses surface electrodes to detect arrhythmias, it is susceptible to inappropriate detection. Although shock conversion rates for the WCD are reported to be high for detected events, its efficacy in clinical practice tends to be degraded by patient noncompliance. Reasons for this include wearer discomfort and frequent false alarms, which may interrupt sleep and generate anxiety. Up to now, data on the incidence of false alarms emitted by the WCD and their predictors are rare. **Objectives:** The aim of our study was to assess the relationship between both artifact sensing and episode misclassification burden and wearing compliance in patients with a WCD (ZOLL LifeVest™ 4000 system, ZOLL CMS GmbH, Cologne, Germany). **Methods and Results:** We conducted a single-center retrospective observational study, analyzing patients with a WCD prescribed at our institution. A total of 134 patients (mean age 51.7 ± 13.8 years, 79.1% male) were included. Arrhythmia recordings were analyzed and categorized as non-sustained ventricular tachycardia, sustained ventricular tachycardia or fibrillation, artifact sensing or misclassified episodes. Indication for WCD prescription was both primary and secondary prophylaxis. A total of 3019 false WCD alarms were documented in 78 patients (average number of false alarms 38.7 ± 169.5 episodes per patient) over a mean WCD wearing time of 71.5 ± 70.9 days (daily WCD wearing time 20.2 ± 5.0 h). In a total of 78 patients (58.2% of the study population), either artifact sensing (76.9%), misclassified episodes (6.4%), or both (16.7%) occurred. Misclassified episodes included sinus tachycardias, atrial flutter, atrial fibrillation, premature ventricular contractions (PVCs), and intermittent bundle branch block. A multiple linear regression identified loop diuretics (regression coefficient [B] −0.11; 95% CI −0.21–(−0.0001); *p* = 0.049), angiotensin receptor–neprilysin inhibitors (ARNIs) (B −0.11; 95% CI 0.22–(−0.01); *p* = 0.033), and a higher R-amplitude of the WCD baseline electrocardiogram (ECG) (B −0.17; 95% CI −0.27–(−0.07); *p* = 0.001) as independent predictors for a lower number of artifact episodes per day. In addition, atrial fibrillation (B 0.05; 95% CI 0.01–0.08; *p* = 0.010), and calcium antagonists (B 0.07; 95% CI 0.02–0.12; *p =* 0.012) were independent predictors for increased numbers of misclassified episodes per day, while beta-blockers seemed to reduce them (B −0.06; 95% CI −0.10–(−0.01); *p* = 0.013). Patients terminated 61.0% of all false alarms manually by pressing the response button on average 1.9 times per false alarm with overall 3.6 manual terminations per affected patient per month. **Conclusions:** In conclusion, false alarms from the ZOLL LifeVest™ system were frequent, with artifact sensing being the most common cause. Hence, the occurrence of false alarms represents a significant side effect of WCD therapy, and efforts should be made to minimize false alarms.

## 1. Introduction

In recent years, wearable cardioverter defibrillator (WCD) therapy has become a useful tool to bridge a temporarily increased risk for sudden cardiac death (SCD), particularly when device implantation is not feasible [1,2,3]. However, although several registries demonstrated that WCDs can prevent SCD in patients at risk [4,5,6], recently published data from the VEST (Vest Prevention of Early Sudden Death) study—the only available randomized controlled trial—showed that being supplied with a WCD does not significantly impact the outcome in patients with myocardial infarction and moderate-to-severe left ventricular systolic dysfunction compared to controls [7]. However, as suggested by a per-protocol analysis of VEST, the efficacy of the WCD may be impacted by patient noncompliance caused by inappropriate shocks, frequent false alarms, and wearing discomfort [8]. Of note, inappropriate shocks may themselves induce ventricular tachycardia or ventricular fibrillation, may cause asystolic cardiac arrest, and often result in emergency room visits or hospitalization.

The current European guidelines governing the prescription of WCDs focus primarily on patients at particularly high SCD risk due to specific clinical conditions. Thus, according to the 2021 European guidelines on heart failure, WCD therapy has a IIb class of recommendation in patients with heart failure who are at high risk for SCD but are not suitable for ICD implantation [9]. The 2022 European guidelines on the management of ventricular arrhythmias recommend the use of WCDs with a IIa class of recommendation for patients with a secondary prevention ICD indication, those who are temporarily not candidates for ICD implantation, and those with a IIb class of recommendation in the early phase after myocardial infarction [10].

Shock conversion rates for WCDs are reported to be high for detected events [5,6], but the efficacy of the WCD in clinical practice is known to be degraded by patient noncompliance. However, data on causal factors of noncompliance, such as the incidence of artifact sensing, misclassified episodes, and their clinical predictors, are rare.

The primary purpose of this study was to assess both the false alarm rate of the LifeVest™ 4000 WCD due to artifact sensing or episode misclassification and patient wearing compliance.

## 2. Methods

### 2.1. Study Design

The study was a single-center retrospective observational trial analyzing patients receiving a WCD between June 2014 and June 2020. Patients were fitted with the current ZOLL LifeVest™ 4000 system (Pittsburgh, PA, USA). The study protocol was approved by the ethics committee (EA 1/356/16) of the Charité, Universitaetsmedizin Berlin and is in accordance with the 1975 Declaration of Helsinki. The study was registered at the German Clinical Trials Register (DRKS00030855).

### 2.2. Study Population

In total, 134 adult patients fitted with a ZOLL LifeVest™ 4000 at our institution were enrolled. The main indication for WCD prescription was a severely impaired left ventricular ejection fraction (LVEF ≤ 35%). Out of 134 patients, 25 patients (18.7%) were prescribed a WCD despite an LVEF > 35% due to high individual risk for sudden cardiac death based on recent history of ventricular tachycardia, ventricular fibrillation, or implantable cardioverter defibrillator (ICD) explantation. All patients received optimal medical therapy according to the current guidelines.

### 2.3. The Wearable Cardioverter Defibrillator

There are currently two systems available: the LifeVest™ 4000 (ZOLL, Pittsburgh, PA, USA) and the ASSURE™ wearable cardioverter defibrillator system (Kestra Medical Technologies^®^, Kirkland, WA, USA). Of those two systems, the LifeVest™ 4000 is the only one available on the European market.

Arrhythmia detection by the LifeVest™ is based on a proprietary algorithm analyzing heart rate and QRS morphology [11]. In the majority of patients, the ventricular tachycardia (VT) threshold was programmed at a heart rate of 150–160 bpm, with a VT response time of 60 s. The ventricular fibrillation (VF) threshold was programmed in the vast majority of cases at a heart rate of 200–210 bpm, with a response time of 25 s in all patients (Supplementary Table S1). The WCD produces a series of warning signals prior to administration of a shock, and patients can press the response button to withhold shock application. The emitted alarms include a silent vibration alert, a loud siren alarm, and a voice command warning bystanders of impending shock administration.

### 2.4. Patient Evaluation and Follow-Up

Baseline evaluation in all patients included transthoracic echocardiography and a 12-lead electrocardiogram. Clinical data, such as medical history, demographics, co-morbidities, and concomitant medical treatment, were collected from the medical records stored in the hospital database.

Stored WCD episodes were classified into the following categories: non-sustained ventricular tachycardias (VTs) (less than 30 s), sustained VTs, ventricular fibrillation (VF), VT/VFs with or without WCD shock therapy, artifact episodes, and misclassified episodes. Data on arrhythmic episodes, artifact sensing, and misclassified episodes were reviewed from the ZOLL Life Vest Network™ and classified by two independent physicians.

### 2.5. Statistical Analysis

Continuous variables are presented as a mean ± SD. Categorical variables are given as absolute and relative frequency. Differences in metric values between independent binary groups were evaluated with a Mann–Whitney U test. Spearman’s correlation was used for bivariate correlation analysis of metric variables. Multiple linear regression was performed with forward and backward selection to identify independent predictors for the number of artifact episodes per day and the number of misclassified episodes per day, respectively. Variables were chosen for inclusion in the multiple regression models based on statistical significance in the univariable regression analyses and clinical judgement. Two-sided *p* values ≤ 0.05 were considered significant. Due to the exploratory characteristics of this study, adjustment for multiplicity was not performed. All statistical analyses were performed with SPSS Statistics Version 29 (IBM Corp., Armonk, NY, USA).

## 3. Results

### 3.1. Baseline Clinical Parameters

We enrolled 134 consecutive patients provided with a WCD at our institution with a mean age of 51.7 ± 13.8 years (79.1% male). Baseline characteristics included age; gender; body mass index; pre-existing heart disease; electrocardiogram (ECG) analysis, including, among others, QRS duration and QTc interval; pre-existing cardiac arrhythmias; and cardiovascular medication (Table 1). Pharmacological antiarrhythmic treatments included beta-blockers (92.5%), amiodarone (7.5%), calcium channel blockers (6.0%), ivabradine (10.4%), and digoxin or digitoxin (3.7%). The indications for WCD use were predominantly ischemic cardiomyopathy (23.9%), dilated cardiomyopathy (34.3%), and inflammatory cardiomyopathy (29.1%). The mean LVEF at baseline was 28.8 ± 12.8%. At the time of WCD prescription, 115 (85.8%) patients were in sinus rhythm, 6 (4.5%) had paroxysmal atrial fibrillation, 11 (8.2%) had persistent atrial fibrillation, 2 (1.5%) had permanent atrial fibrillation, and 5 (3.7%) showed first-degree atrioventricular (AV) block. Two patients were implanted with a conventional transvenous pacemaker and one with a leadless pacemaker (Micra™ VR; Medtronic, Minneapolis, MN, USA). Of note, 61 (45.5%) patients had a history of ventricular arrhythmia at the time of WCD prescription, including non-sustained ventricular tachycardia (*n* = 46; 34.3% of the total study population), sustained ventricular tachycardia (*n* = 4; 3.0%), and ventricular fibrillation (*n* = 13; 9.7%).

### 3.2. Wearable Cardioverter Defibrillator Use and Wearing Compliance

**Figure 1:**
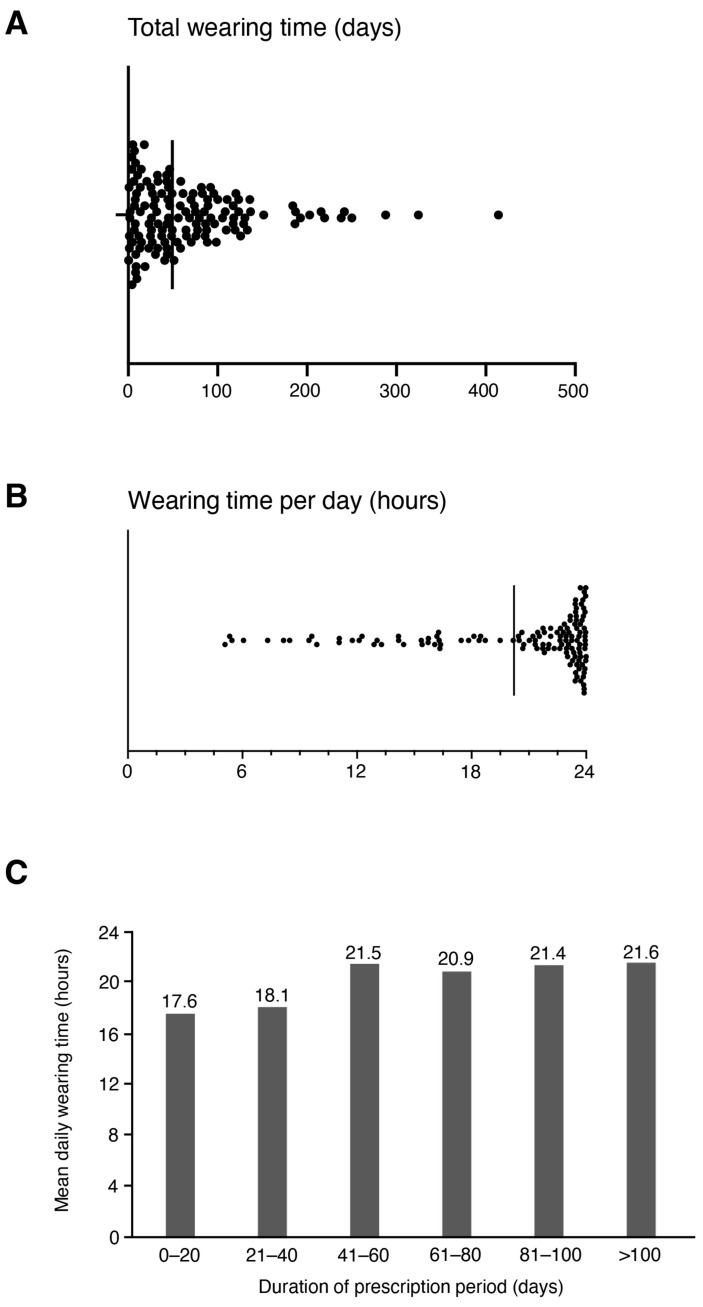
Distribution of WCD wearing time. (**A**) Total WCD wearing time in days. (**B**) WCD wearing time per day (hours). Vertical lines denote median values. (**C**) Mean daily WCD wearing time (hours) depending on the prescription period in days.

The mean WCD wearing time was 71.5 ± 70.9 days, with a mean WCD wearing time per day of 20.2 ± 5.0 h (Figure 1A,B). The mean WCD wearing time and wearing time per day were comparable in both females (20.7 ± 4.2 h per day) and males (20.1 ± 5.2 h per day; *p* = 0.81). The majority of patients (*n* = 78, 58.2%) wore the WCD between 22 and 24 h per day. However, we observed significantly lower daily WCD wearing times in patients <55 years compared to patients ≥55 years (19.4 ± 5.6 vs. 21.1 ± 4.2 h per day, *p* = 0.019). The daily WCD wearing time showed no correlation with the prescription duration (*p* = 0.113) (Figure 1C).

### 3.3. Wearable Cardioverter Defibrillator Arrhythmia Episodes

**Figure 2:**
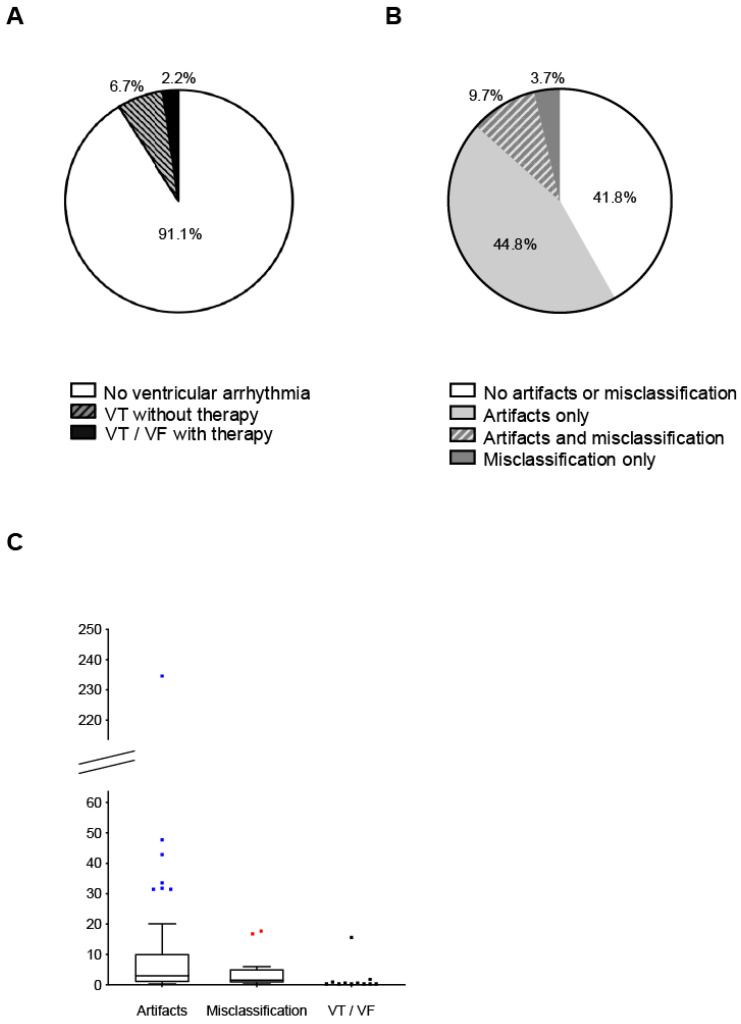
(**A**) Percentage of patients with ventricular arrhythmias with and without shock delivery. (**B**) Percentage of patients with artifact sensing and/or episode misclassification. (**C**) Absolute number of patients with artifact sensing, episode misclassification, and ventricular arrhythmias. VT, ventricular tachycardia; VF, ventricular fibrillation.

Sustained ventricular tachycardia (sVT) occurred in five patients (9 sVTs in total), non-sustained ventricular tachycardia (nsVT) occurred in seven patients (13 nsVTs in total), and ventricular fibrillation occurred in one patient (Figure 2A). A total of three patients, two with ischemic cardiomyopathy and one with inflammatory cardiomyopathy, received appropriate WCD shocks. In one patient, the delivery of two WCD shocks was required to terminate the ventricular tachycardia (Supplementary Table S2).

Overall, WCD was prematurely discontinued in ten patients. The reasons for discontinuation were known in six cases and included discomfort, frequent false alarms, and fear of being mistaken for an assassin. One patient died during the WCD prescription period after taking off the WCD to shower. Another patient died of sudden cardiac death after he stopped wearing the WCD due to frequent artifact episodes.

### 3.4. False Wearable Cardioverter Defibrillator Recordings

A total of 3019 false WCD alarms occurred in 78 (58.2% of the study population) patients during the observation period due to either artifact sensing or episode misclassification (Table 2). The number of false alarms correlated positively with the daily WCD wearing time (correlation coefficient = 0.247, *p =* 0.004). Overall, 2917 artifact episodes occurred in a total of 73 patients (93.6% of all patients with false alarms) and 102 episodes misclassifications occurred in 18 patients (23.1% of all patients with false alarms). Each of these patients experienced, on average, 37.4 ± 169.6 alarms due to artifact episodes and 1.3 ± 3.7 alarms due to misclassified episodes.

Misclassified episodes included supraventricular tachycardias (sinus tachycardia, atrial fibrillation, atrial flutter), rate-controlled atrial fibrillation, premature ventricular contractions (PVCs), and intermittent bundle branch block (Supplementary Table S3). In 60 (44.8% of all study patients) patients, only artifact sensing episodes occurred; in 5 (3.7%) patients, only episode misclassification occurred; and in 13 (9.7%) patients, both artifact sensing and misclassification were recorded (Figure 2B). The mean number of false alarms due to artifact sensing or episode misclassification was 6.1 ± 21.6 alerts per affected patient per month compared to 0.28 ± 2.73 appropriate alerts per patient per month due to ventricular tachycardia or ventricular fibrillation (Figure 2C). In total, 1177 (39.0%) of all false alarms were automatically terminated.

### 3.5. Manual Termination of False Alarms by the Patient

**Figure 3:**
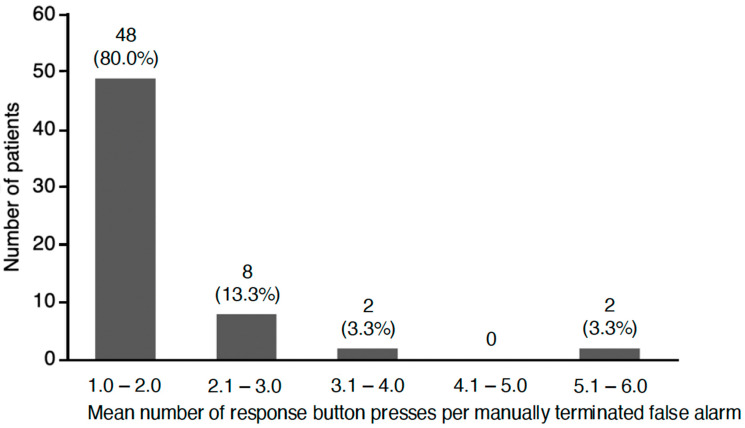
Mean number of times the WCD response button was pressed by the patient per manually terminated false alarm to prevent shock delivery. Values are given as *n* (% of the subpopulation who manually terminated false alarms; total *n* = 60).

**Figure 4:**
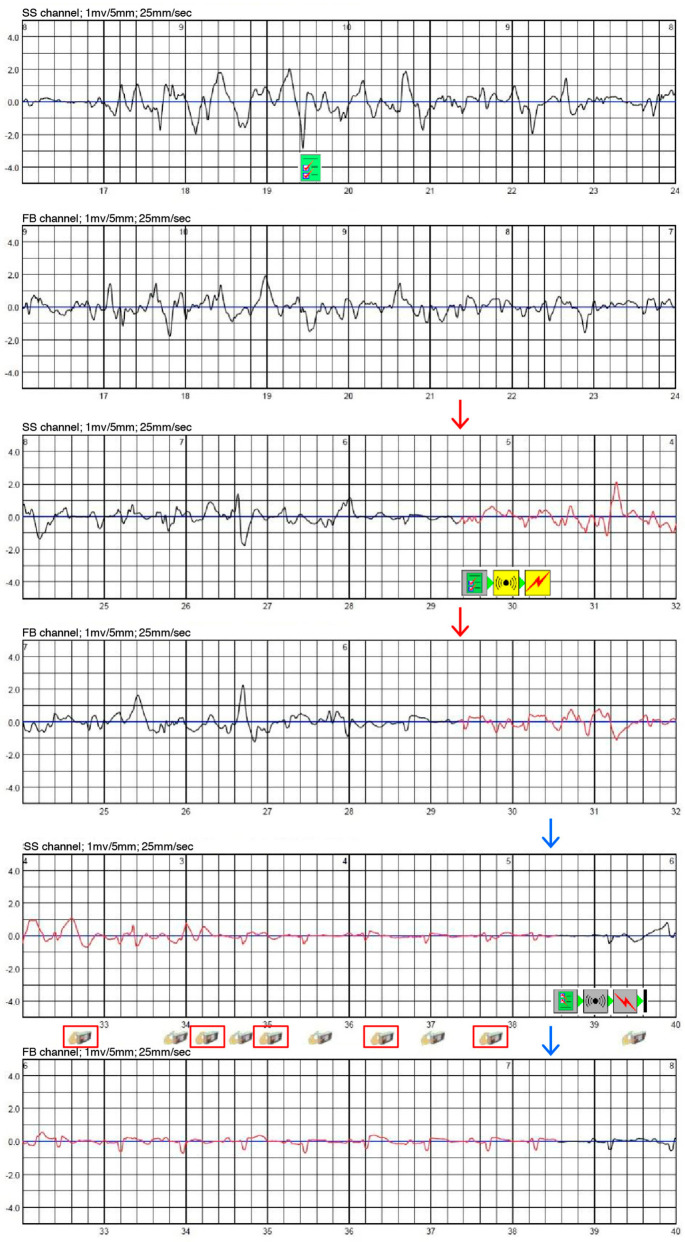
Exemplary ECG recording of motion-related artifact sensing that leads to the initiation of the WCD alarm sequence. Red arrows mark the beginning and blue arrows mark the end of the WCD alert. 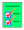: Arrhythmia is validated. 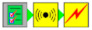: Start of WCD treatment sequence. 
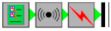
: End of treatment sequence. 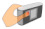: Response button was pressed. 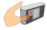: Response button was released.

Overall, 1842 (61.0%) false alarms were manually terminated by patients (including 61.2% of all artifact episodes and 55.9% of all misclassified episodes). On average, there were 3.6 false alarms manually terminated per affected patient per month (see Table 2). Patients who manually terminated false alarms pressed the response button, on average, 1.9 times per false alarm (Figure 3). Figure 4 shows an example of multiple presses of the response button due to a false alarm.

### 3.6. Variables Associated with Wearable Cardioverter Defibrillator Artifact Sensing and Episode Misclassification

In the univariable linear regression analyses, loop diuretics, angiotensin receptor–neprilysin inhibitors (ARNIs), QTc time in the 12-lead ECG, and R-wave amplitude in the baseline WCD ECG were significantly associated with the number of artifact episodes per day (Table 3A). The multiple linear regression identified loop diuretics, ARNIs, and the R-wave amplitude of baseline WCD ECG as independent predictors of the number of artifact episodes per day. Moreover, in the univariable linear regression analyses, calcium channel antagonists, beta-blockers, and atrial fibrillation significantly correlated with the number of misclassified episodes per day. These variables remained independent predictors in the multiple linear regression for the number of misclassified episodes per day (Table 3B).

## 4. Discussion

The main findings of our study of a real-world patient population compromising different indications for WCD prescription are that (1) artifact sensing occurred in more than half of the WCD patients; (2) misclassification of episodes was noted in approximately 13% of the WCD patients; (3) loop diuretics, ARNIs, and the R-wave amplitude in the baseline WCD ECG were significantly associated with the artifact episode number per day; (4) calcium channel blockers, beta-blockers, and atrial fibrillation significantly correlated with the number of misclassified episodes per day; and (5) around 60% of all false alarms were terminated manually by the patient (Supplementary Figure S1).

Previous studies have reported the occurrence of inappropriate arrhythmia detection and inappropriate shock delivery in various WCD patient populations [12,13,14]. In our cohort, false WCD alarms, mainly due to artifact sensing, were common. Loop diuretics, ARNIs, and the R-wave amplitude of baseline WCD ECGs were identified as independent predictors of the number of artifact episodes per day. Reduced sweat production due to loop diuretic intake and the diuretic potential of ARNIs are possible explanations for the lower frequency of artifact sensing [15]. High R-wave amplitudes on the WCD ECG show that a well-fitted garment with good ECG electrode contact is important for effective noise immunity. With regard to the number of misclassified episodes per day, calcium channel antagonists, beta-blockers, and atrial fibrillation were significantly correlated with the number of misclassified episodes. One possible explanation for the lower incidence of misclassified episodes with beta-blocker use is their frequency-limiting effect. On the other hand, increased sweat production due to calcium channel antagonist intake and an associated decrease in the R-wave amplitude of the WCD ECG are possible reasons for the increase in misclassified episodes. However, false alarms due to either artifact sensing or episode misclassification do not appear to have had a negative effect on the patients’ daily WCD wearing time in our study population. In contrast to the findings from the Swiss WCD registry [13], obesity did not correlate with the rate of false alarm burden in our study patients. An increased movement of the subcutaneous fatty tissue in relation to the heart, in the case of severe obesity, may cause an increased rate of alarm burden. However, the lower BMI (27.7 ± 6.9) in our study cohort compared to those in the retrospective Swiss WCD registry may explain why the BMI did not correlate with the false alarm rate in our study population. Other patient-specific baseline characteristics, such as sex or age, did not show a statistically significant correlation with the incidence of false WCD alarms. Poole et al. recently reported that a new WCD (ASSURE WCD system, Kestra Medical Technologies, Inc., Kirkland, WA, USA) has lower false alarm rates than those reported for the LifeVest™ 4000 [16]. However, the LifeVest™ 4000 is currently the only WCD available on the European market.

In our study cohort, ventricular arrhythmias (non-sustained or sustained ventricular tachycardias) occurred in 8.2% of all patients and ventricular fibrillation in one patient. We report an overall incidence of appropriate shock delivery of 3.0 per 100 persons over 3 months, which was lower than in a previously published meta-analysis, which reported a pooled incidence from all studies of 5 per 100 persons over 3 months [4]. Moreover, Masri et al. reported an incidence of inappropriate WCD therapy of 2 per 100 persons over 3 months. However, in their meta-analysis, Masri et al. reported a large range in treatment incidences and also significant heterogeneity among the included studies with regard to indications for WCD use. Of note, no inappropriate WCD shock release occurred in our study population. This is in accordance with previous studies reporting a low percentage of WCD patients receiving inappropriate shocks [7,17,18]. Overall, the prescription rate of amiodarone (*n* = 10; 7.5%) was low in our study cohort. Reasons for this include the young age of our study population (51.7 ± 13.8 years) and concerns of the patients regarding possible side effects.

The alarm sequence of WCDs initiated before shock delivery is important due to the limited reliability of surface ECG recordings. The sequence of vibratory and auditory alarms emitted by the device enables the patient to press a response button to abort inadequate shock delivery. However, false alarms that require the patient to abort shock therapy have a great significance, as they have deleterious effects on patients’ quality of life and may impact adherence to the WCD therapy [19]. In our study cohort, the response button was pressed at least once in 61.2% of all artifact episodes and 55.9% of all misclassified episodes to withhold WCD shock delivery. Episodes of frequent manual deactivation often led to stress-induced sinus tachycardia in the course of the WCD deactivations.

Overall, WCD adherence was high in our study population, with an average daily wear time of 20.2 ± 5.0 h, which is comparable to previous findings from large observational trials [4,5,20]. However, despite frequent false alarms, no negative impact on the daily WCD wearing time was observed in our study population. With regard to the effect of prescription duration on the daily WCD wearing time, previous studies have shown varying results [14,17]. In our study cohort, WCD wearing compliance was independent of the WCD prescription period, but significantly higher in the group of patients aged 55 years or older. This corresponds to previous studies reporting lower daily WCD wearing compliance in younger patients [5,14].

Several studies, including two registry trials, demonstrated the effectiveness of WCDs [5,17,18,21]. However, the VEST trial, as currently the only randomized WCD trial so far, failed to demonstrate a benefit with regard to the prevention of arrhythmic death in high-risk patients after myocardial infarction [7]. A central limitation in this trial was poor WCD wearing compliance with a median of only 18 h per day and frequent premature interruption of the WCD therapy. The WCD wearing time in the VEST trial was significantly lower than in preceding registry studies, with median daily uses of 23.1 h and 22.5 h, respectively [5,17]. A crucial factor in the effectiveness of the WCD is adequate patient compliance. In addition to detailed training provided by the manufacturer at the time of WCD delivery, standardized patient training programs are a promising approach to further increase WCD adherence [22].

To date, only small retrospective studies on the quality of life of patients with WCD use have been published. Lackermair et al. [19] reported that WCD use was associated with a high rate of sleep disturbances and fear of shock, but also feelings of security. In our study, the frequency of artifact sensing occurrence and episode misclassification had no negative impact on the average daily WCD wearing time. However, ten of our patients discontinued WCD use prematurely, of which two were due to the occurrence of false alarms. Previous studies have shown that standardized training and adherence surveillance programs might have beneficial effects on the quality of life of WCD patients [23].

## 5. Conclusions

With the use of WCDs, false alarms caused by either artifact sensing or episode misclassification are frequent and a majority must be manually terminated by the patient. Due to the fact that WCD therapy is only effective if the vest is worn, attention needs to be given to patients with a high burden of false alarms. Although we did not observe decreased WCD therapy adherence depending on the number of false alarms, the impact of false alarms on the quality of life should not be underestimated. Thus, keeping an eye on the frequency of false alarms should be an integral part of the clinical routine for WCD patients, and every effort should be made to minimize the frequency of their occurrence.

## Figures and Tables

**Table 1:** Baseline characteristics of the patient population.

Baseline Characteristics of the Patient Population	Total Population (*n* = 134) *n* (%) or Mean ± SD
Demographics and clinical data	
Age (years)	51.7 ± 13.8
Male gender	106 (79.1%)
BMI (kg/m^2^)	27.7 ± 6.9
Systolic blood pressure (mmHg)	118.0 ± 18.2
Diastolic blood pressure (mmHg)	73.0 ± 15.4
WCD indication	
Primary prophylaxis	73 (54.5%)
Secondary prophylaxis	61 (45.5%)
Pre-existing heart disease	
Ischemic cardiomyopathy	32 (23.9%)
Dilated cardiomyopathy	47 (34.3%)
Inflammatory cardiomyopathy	39 (29.1%)
Hypertrophic cardiomyopathy	3 (2.2%)
Non-compaction cardiomyopathy	2 (1.5%)
Tako-Tsubo cardiomyopathy	1 (0.7%)
Valvular cardiomyopathy	2 (1.5%)
PVC-induced cardiomyopathy	1 (0.7%)
Specific cardiomyopathy not clearly assignable	7 (5.2%)
Pre-existing cardiac arrhythmias	
Ventricular tachycardia	50 (37.3%)
Non-sustained ventricular tachycardia	46 (34.3%)
Sustained ventricular tachycardia	4 (3.0%)
Ventricular fibrillation	13 (9.7%)
Atrial fibrillation	19 (14.2%)
Paroxysmal atrial fibrillation	6 (4.5%)
Persistent atrial fibrillation	11 (8.2%)
Permanent atrial fibrillation	2 (1.5%)
Left bundle branch block	31 (23.1%)
Right bundle branch block	8 (6.0%)
Atrioventricular block °I °III	5 (3.7%) 1 (0.7%)
Cardiovascular medication	
ACE inhibitors	65 (48.5%)
ARBs	56 (41.8%)
Beta-blockers	124 (92.5%)
Loop diuretics	98 (73.1%)
MRAs	63 (47.0%)
ARNIs	39 (29.1%)
Digoxin/Digitoxin	5 (3.7%)
Amiodarone	10 (7.5%)
Ivabradine	14 (10.4%)
Calcium channel blockers	8 (6.0%)
12-lead ECG	
HR (bpm)	80.0 ± 17.5
PQ (ms)	166.9 ± 32.0
QRS (ms)	117.7 ± 31.3
QTc (ms)	456.5 ± 44.0
ST elevation	13 (9.7%)
ST depression	5 (3.7%)
T wave inversion	35 (26.1%)
LifeVest 2-lead ECG	
QRS (ms)	118.2 ± 28.7
R-amplitude (mV)	1.1 ± 0.5
Echocardiographic parameters	
LVEF (%)	28.8 ± 12.8
LVEDD (mm)	60.8 ± 9.9

Values are presented as *n* (% of the total study population) or a mean ± standard deviation (SD). ACE, angiotensin converting enzyme; ARB, angiotensin receptor blocker; ARNI, angiotensin receptor–neprilysin inhibitor; ECG, electrocardiogram; LVEDD, left ventricular end diastolic diameter; LVEF, left ventricular ejection fraction; MRA, mineralocorticoid receptor antagonist; PVC, premature ventricular complex; WCD, wearable cardioverter defibrillator.

**Table 2:** Incidence of false alarms due to artifact sensing and episode misclassification.

False Alarms (Including Artifact and Misclassified Episodes)	*n* (%) or Mean ± SD
Number of patients with false alarms	78 (58.2%)
Total number of false alarms	3019
Average number of false alarms per affected patient	38.7 ± 169.5
Average number of false alarms per affected patient per month	6.1 ± 21.6
Total number of automatically terminated false alarms	1177 (39.0%)
Average number of automatically terminated false alarms per affected patient	15.1 ± 63.8
Average number of automatic terminations per affected patient per month	2.5 ± 8.6
Total number of manually terminated false alarms	1842 (61.0%)
Average number of manually terminated false alarms per affected patient	23.6 ± 106.9
Average number of manual terminations per affected patient per month	3.6 ± 13.5
**Artifact episodes**	
Number of patients with artifact episodes	73 (93.6%)
Total number of artifact episodes	2917
Average number of artifact episodes per affected patient	37.4 ± 169.6
Average number of artifact episodes per affected patient per month	5.6 ± 21.6
**Misclassified episodes**	
Number of patients with misclassified episodes	18 (23.1%)
Total number of misclassified episodes	102
Average number of misclassified episodes per affected patient	1.3 ± 3.7
Average number of misclassified episodes per day per affected patient	0.5 ± 2.2

Values are presented as *n* (% of the total population or subcategory) or a mean ± standard deviation (SD).

**Table 3:** Univariable and multiple linear regression analyses of the number of artifact episodes (**A**) and misclassified episodes (**B**) per day.

A. Clinical Predictors for Artifact Sensing						
**Variable**	**Univariable Linear Regression**			**Multiple Linear Regression**		
	Regression coefficient B	95% CI	*p* value	Regression coefficient B	95% CI	*p* value
R-wave amplitude (WCD ECG)	−0.177	−0.276–(−0.078)	0.001	−0.171	−0.274–(−0.068)	0.001
Loop diuretics	−0.165	−0.269–(−0.061)	0.002	−0.107	−0.214–(−0.0001)	0.0498
ARNIs	−0.126	−0.229–(−0.023)	0.017	−0.112	−0.215–(−0.009)	0.033
**B. Clinical Predictors for Misclassified Episodes**						
**Variable**	**Univariable Linear Regression**			**Multiple Linear Regression**		
	Regression coefficient B	95% CI	*p* value	Regression coefficient B	95% CI	*p* value
Atrial fibrillation	0.047	0.011–0.083	0.01	0.046	0.011–0.081	0.01
Beta-blockers	−0.069	−0.116–(−0.022)	0.004	−0.058	−0.104–(−0.012)	0.013
Calcium antagonists	0.062	0.009–0.115	0.022	0.066	0.015–0.117	0.012

For statistical reasons, one outlier patient with a total of 1438 artifact episodes was excluded. ARNI, angiotensin receptor–neprilysin inhibitor; CI, confidence interval; ECG, electrocardiogram; WCD, wearable cardioverter defibrillator.

## Data Availability

The participants of this study did not give written consent for their data to be shared publicly.

## Supplementary Materials

The following supporting information can be downloaded at: https://www.mdpi.com/article/10.3390/jcm13247768/s1, Figure S1: Study design flow chart, Table S1: WCD programming of the study population, Table S2: Incidence and type of ventricular arrhythmias, Table S3: Types of misclassified episodes.

## References

[1] Lenormand T., Bodin A., Fauchier L. (2024). The Role of the Wearable Defibrillator in Heart Failure. Curr. Heart Fail. Rep..

[2] Rohrer U., Manninger M., Fiedler L., Steinwender C., Binder R.K., Stuhlinger M., Zirngast B., Zweiker D., Zirlik A., Scherr D. (2023). Prevention of Early Sudden Cardiac Death after Myocardial Infarction Using the Wearable Cardioverter Defibrillator-Results from a Real-World Cohort. J. Clin. Med..

[3] Blaschke F., Lacour P., Dang P.L., Parwani A.S., Hohendanner F., Walter T., Klingel K., Kuhl U., Heinzel F.R., Sherif M. (2021). Wearable cardioverter-defibrillator: Friend or foe in suspected myocarditis?. ESC Heart Fail..

[4] Masri A., Altibi A.M., Erqou S., Zmaili M.A., Saleh A., Al-Adham R., Ayoub K., Baghal M., Alkukhun L., Barakat A.F. (2019). Wearable Cardioverter-Defibrillator Therapy for the Prevention of Sudden Cardiac Death: A Systematic Review and Meta-Analysis. JACC Clin. Electrophysiol..

[5] Wassnig N.K., Gunther M., Quick S., Pfluecke C., Rottstadt F., Szymkiewicz S.J., Ringquist S., Strasser R.H., Speiser U. (2016). Experience With the Wearable Cardioverter-Defibrillator in Patients at High Risk for Sudden Cardiac Death. Circulation.

[6] Ellenbogen K.A., Koneru J.N., Sharma P.S., Deshpande S., Wan C., Szymkiewicz S.J. (2017). Benefit of the Wearable Cardioverter-Defibrillator in Protecting Patients After Implantable-Cardioverter Defibrillator Explant: Results from the National Registry. JACC Clin. Electrophysiol..

[7] Olgin J.E., Pletcher M.J., Vittinghoff E., Wranicz J., Malik R., Morin D.P., Zweibel S., Buxton A.E., Elayi C.S., Chung E.H. (2018). Wearable Cardioverter-Defibrillator after Myocardial Infarction. N. Engl. J. Med..

[8] Olgin J.E., Lee B.K., Vittinghoff E., Morin D.P., Zweibel S., Rashba E., Chung E.H., Borggrefe M., Hulley S., Lin F. (2020). Impact of wearable cardioverter-defibrillator compliance on outcomes in the VEST trial: As-treated and per-protocol analyses. J. Cardiovasc. Electrophysiol..

[9] McDonagh T.A., Metra M., Adamo M., Gardner R.S., Baumbach A., Bohm M., Burri H., Butler J., Celutkiene J., Chioncel O. (2021). 2021 ESC Guidelines for the diagnosis and treatment of acute and chronic heart failure. Eur. Heart J..

[10] Zeppenfeld K., Tfelt-Hansen J., de Riva M., Winkel B.G., Behr E.R., Blom N.A., Charron P., Corrado D., Dagres N., de Chillou C. (2022). 2022 ESC Guidelines for the management of patients with ventricular arrhythmias and the prevention of sudden cardiac death. Eur. Heart J..

[11] Reek S., Burri H., Roberts P.R., Perings C., Epstein A.E., Klein H.U., Committee E.S.D., Lip G., Gorenek B., Sticherling C. (2017). The wearable cardioverter-defibrillator: Current technology and evolving indications. Europace.

[12] Berger J.M., Sengupta J.D., Bank A.J., Casey S.A., Witt D., Sharkey S.W., Stanberry L.I., Hauser R.G. (2023). Causes and clinical consequences of inappropriate shocks experienced by patients wearing a cardioverter-defibrillator. Heart Rhythm..

[13] Kovacs B., Burri H., Buehler A., Reek S., Sticherling C., Schaer B., Linka A., Ammann P., Muller A.S., Dzemali O. (2021). High Incidence of Inappropriate Alarms in Patients with Wearable Cardioverter-Defibrillators: Findings from the Swiss WCD Registry. J. Clin. Med..

[14] Zylla M.M., Hillmann H.A.K., Proctor T., Kieser M., Scholz E., Zitron E., Katus H.A., Thomas D. (2018). Use of the wearable cardioverter-defibrillator (WCD) and WCD-based remote rhythm monitoring in a real-life patient cohort. Heart Vessels.

[15] Wang T.D., Tan R.S., Lee H.Y., Ihm S.H., Rhee M.Y., Tomlinson B., Pal P., Yang F., Hirschhorn E., Prescott M.F. (2017). Effects of Sacubitril/Valsartan (LCZ696) on Natriuresis, Diuresis, Blood Pressures, and NT-proBNP in Salt-Sensitive Hypertension. Hypertension.

[16] Poole J.E., Gleva M.J., Birgersdotter-Green U., Branch K.R.H., Doshi R.N., Salam T., Crawford T.C., Willcox M.E., Sridhar A.M., Mikdadi G. (2022). A wearable cardioverter defibrillator with a low false alarm rate. J. Cardiovasc. Electrophysiol..

[17] Kutyifa V., Moss A.J., Klein H., Biton Y., McNitt S., MacKecknie B., Zareba W., Goldenberg I. (2015). Use of the wearable cardioverter defibrillator in high-risk cardiac patients: Data from the Prospective Registry of Patients Using the Wearable Cardioverter Defibrillator (WEARIT-II Registry). Circulation.

[18] Garcia R., Combes N., Defaye P., Narayanan K., Guedon-Moreau L., Boveda S., Blangy H., Bouet J., Briand F., Chevalier P. (2021). Wearable cardioverter-defibrillator in patients with a transient risk of sudden cardiac death: The WEARIT-France cohort study. Europace.

[19] Lackermair K., Schuhmann C.G., Kubieniec M., Riesinger L.M., Klier I., Stocker T.J., Kaab S., Estner H.L., Fichtner S. (2018). Impairment of Quality of Life among Patients with Wearable Cardioverter Defibrillator Therapy (LifeVest(R)): A Preliminary Study. BioMed Res. Int..

[20] Kovacs B., Reek S., Sticherling C., Schaer B., Linka A., Ammann P., Brenner R., Krasniqi N., Muller A.S., Dzemali O. (2020). Use of the wearable cardioverter-defibrillator—The Swiss experience. Swiss Med. Wkly..

[21] Chung M.K., Szymkiewicz S.J., Shao M., Zishiri E., Niebauer M.J., Lindsay B.D., Tchou P.J. (2010). Aggregate national experience with the wearable cardioverter-defibrillator: Event rates, compliance, and survival. J. Am. Coll. Cardiol..

[22] Fazzini L., Marchetti M.F., Perra F., Biddau M., Massazza N., Nissardi V., Agus E., Demelas R., Montisci R. (2023). Does Patient Compliance Influence Wearable Cardioverter Defibrillator Effectiveness? A Single-Center Experience. J. Clin. Med..

[23] Kellnar A., Fichtner S., Sams L., Stremmel C., Estner H.L., Lackermair K. (2023). Evaluation of a Standardized Training and Adherence Surveillance Programme to Overcome Quality-of-Life Impairments and Enhance Compliance in Patients Treated with Wearable Cardioverter Defibrillator. Patient Prefer. Adher..

