# Supplementary material for: False Alarms in Wearable Cardioverter Defibrillators—A Relevant Issue or an Insignificant Observation
Source: J Clin Med. 2024 Dec 19;13(24):7768. doi: 10.3390/jcm13247768 (PMC11728023; doi:10.3390/jcm13247768)
Supplement: Supplementary file 1 [file jcm-13-07768-s001.zip › jcm-3331862-supplementary.pdf]

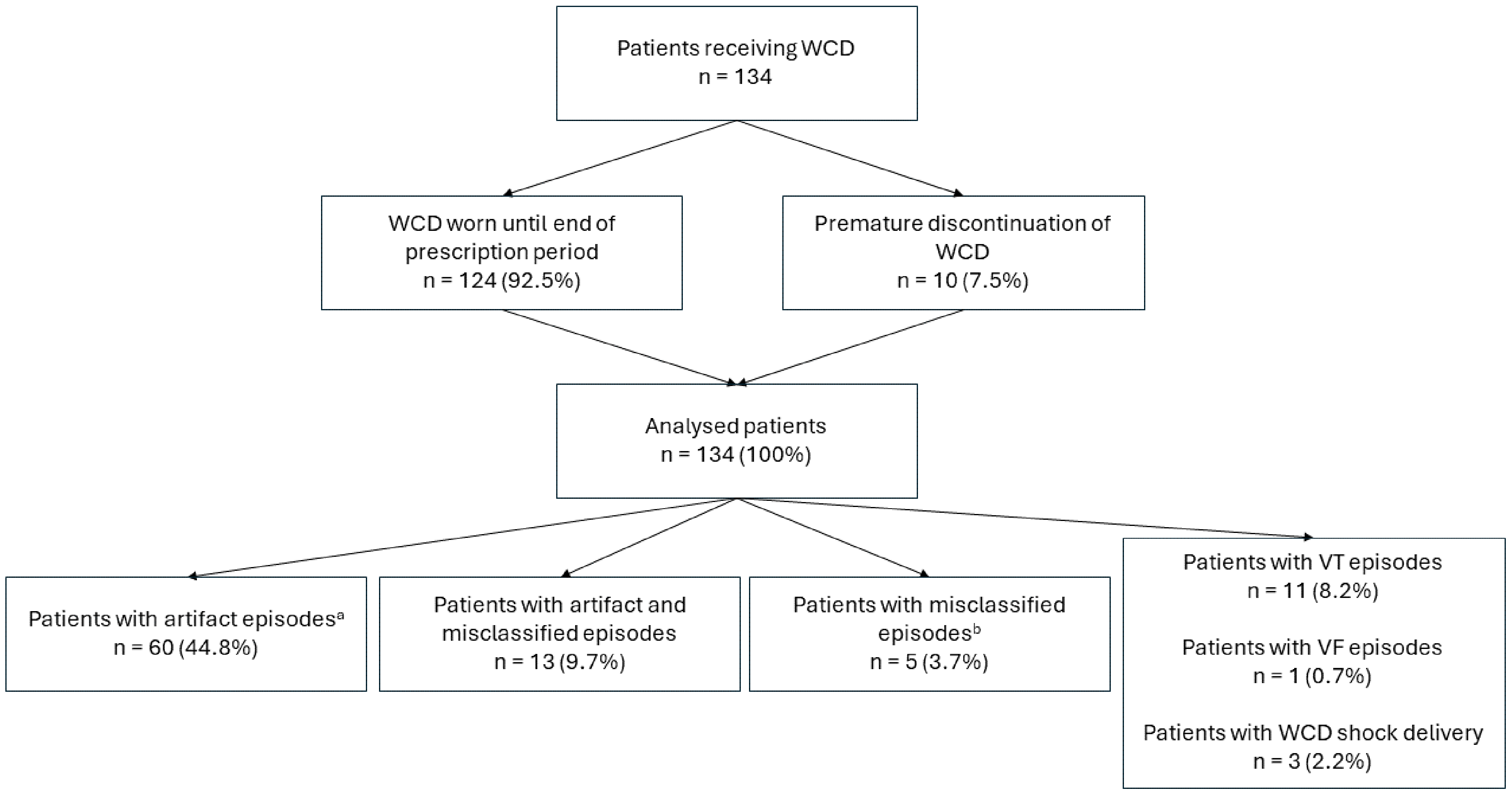

Supplemental Figure S1: Study design low chart

Values are presented as n (% of the total study population). WCD, wearable cardioverter defibrillator; VT, ventricular tachycardia; VF, ventricular fibrillation. a: without misclassified episodes, b: without artifact episodes.

Supplemental Table S1: WCD programming of the study population

**Ventricular tachycardia (VT) zone**		
**Thresholds (bpm)**	**n**	**%**
140	1	0.8
150 - 160	115	87.1
170 - 190	14	10.6
≥ 200	2	1.5
**Response Time (sec)**		
60	131	99.2
90	1	0.8

**Ventricular fibrillation (VF) zone**		
**Thresholds (bpm)**	**n**	**%**
190	1	0.8
200 - 210	126	95.5
≥ 220	5	3.8
**VF Response Time (sec)**		
25	132	100

Values are presented as n (% of the total study population). bpm, beats per minute.

Supplemental Table S2: Incidence and type of ventricular arrhythmias

**Episodes of Ventricular Tachycardia and Ventricular Fibrillation**	**n (%)**
Patients with VT episodes	11 (8.2%)
VT episodes	22
sustained VT episodes	9
patients with sustained VT	5 (3.7%)
non-sustained VT episodes	13
patients with non-sustained VT	7 (5.2%)
Patients with VF episodes	1 (0.7%)
VF episodes	1
Patients with WCD shock delivery	3 (2.2%)
Total number of WCD shocks	4
due to VF episodes	1
due to VT episodes	3

Values are given as n (% of the total population), VT, ventricular tachycardia; VF, ventricular fibrillation.

Supplemental Table S3: Types of misclassified episodes

**Types of misclassified episodes**	n (%)
Sinus tachycardia	38 (37.3%)
Tachycardic atrial fibrillation	38 (37.3%)
Normofrequent atrial fibrillation	3 (2.9%)
Atrial flutter	21 (20.6%)
Premature ventricular contractions	1 (0.9%)
Intermittent bundle branch block	1 (0.9%)

Values are given as n (% of the total population).
